# Editorial Comment: Management of large renal stones with super-mini percutaneous nephrolithotomy: an international multicentre comparative study

**DOI:** 10.1590/S1677-5538.IBJU.2021.05.05

**Published:** 2021-07-23

**Authors:** Fábio C. M. Torricelli

**Affiliations:** 1 Universidade de São Paulo Faculdade de Medicina Hospital das Clínicas São PauloSP Brasil Divisão de Urologia do Hospital das Clínicas da Faculdade de Medicina da Universidade de São Paulo, São Paulo, SP, Brasil

Yang Liu^1^, Chao Cai^1^, Albert Aquino^2^, Shabir Al-Mousawi^3^, Xuepei Zhang^4^, Simon K S Choong^5^, et al.

BJU Int. 2020 Jul;126(1):168-176.

## COMMENT

Percutaneous nephrolithotomy (PCNL) is the gold standard surgical treatment for large renal kidney stones (>2.0 cm) according to EUA and AUA guidelines (1, 2). However, this procedure is related to a higher morbidity (longer hospital stay and higher blood loss) when compared to retrograde intrarenal surgery (RIRS). Improvements and miniaturization of surgical devices with enriched technology aim to reach the optimal stone clearance with the lowest complication rate. Liu et al. in this retrospective multi-center study comparing 2 groups with 1380 matched patients with kidney stones > 2.0 cm showed that super mini-PCNL (8 F nephroscope; 12 or 14 F sheath; 0.8 mm pneumatic lithotripter or 550 μm fiber) has lower perioperative hemoglobin drop, shorter hospital stay, lower postoperative pain score with higher tubeless rate, reaching a similar stone-free rate when compared to mini-PCNL. Moreover, for stones raging from 2 to 3 cm, super mini-PCNL had a higher stone-free rate. In another study, when compared to standard PCNL for treatment of kidney stones up to 2,0 cm, super mini-PCNL achieved equal stone-free rate, whereas had a shorter hospital, and lower incidence of bleeding and postoperative pain (3).

Several authors have already reported favorable outcomes of mini-PCNL when compared to RIRS (4-6). A similar or a higher stone-free rate is the main advantage. For lower pole kidney stone this advantage of mini-PCNL is even clearer. Certainly, miniaturization and incorporation of new technologies will be the future of endourology and will help urologists to achieve better postoperative outcomes.
